# Atypical ductal hyperplasia: update on diagnosis, management, and molecular landscape

**DOI:** 10.1186/s13058-018-0967-1

**Published:** 2018-05-02

**Authors:** Tanjina Kader, Prue Hill, Emad A. Rakha, Ian G. Campbell, Kylie L. Gorringe

**Affiliations:** 1 Cancer Genetics Laboratory, Peter MacCallum Cancer Centre, Melbourne, VIC Australia; 2 The Sir Peter MacCallum Department of Oncology, University of Melbourne, Melbourne, VIC Australia; 3 Cancer Genomics Program, Peter MacCallum Cancer Centre, Melbourne, VIC Australia; 4 Department of Anatomical Pathology, St Vincent’s Hospital, Fitzroy, VIC Australia; 5 Department of Histopathology, University of Nottingham and Nottingham University Hospitals NHS Trust, City Hospital, Nottingham, UK; 6 Department of Pathology, University of Melbourne, Parkville, VIC Australia

**Keywords:** Atypical ductal hyperplasia, Breast neoplasms, Ductal carcinoma in situ, Breast cancer progression, Clonal relationship, Patient care management

## Abstract

**Background:**

Atypical ductal hyperplasia (ADH) is a common diagnosis in the mammographic era and a significant clinical problem with wide variation in diagnosis and treatment. After a diagnosis of ADH on biopsy a proportion are upgraded to carcinoma upon excision; however, the remainder of patients are overtreated. While ADH is considered a non-obligate precursor of invasive carcinoma, the molecular taxonomy remains unknown.

**Main text:**

Although a few studies have revealed some of the key genomic characteristics of ADH, a clear understanding of the molecular changes associated with breast cancer progression has been limited by inadequately powered studies and low resolution methodology. Complicating factors such as family history, and whether the ADH present in a biopsy is an isolated lesion or part of a greater neoplastic process beyond the limited biopsy material, make accurate interpretation of genomic features and their impact on progression to malignancy a challenging task. This article will review the definitions and variable management of the patients diagnosed with ADH as well as the current knowledge of the molecular landscape of ADH and its clonal relationship with ductal carcinoma in situ and invasive carcinoma.

**Conclusions:**

Molecular data of ADH remain sparse. Large prospective cohorts of pure ADH with clinical follow-up need to be evaluated at DNA, RNA, and protein levels in order to develop biomarkers of progression to carcinoma to guide management decisions.

## Background

**Fig. 1:**
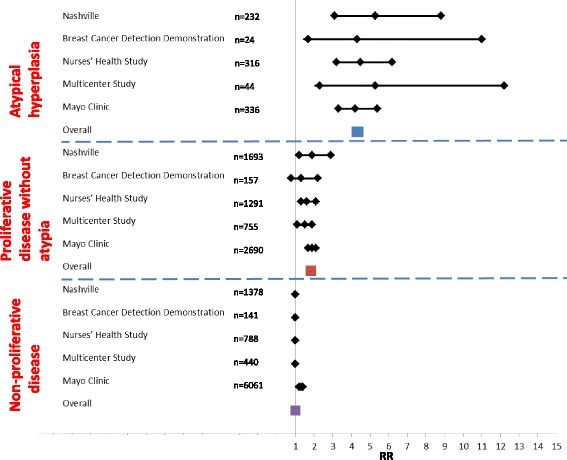
Forest plot showing histologic category of benign breast lesions and relative risk for breast cancer with 95% confidence interval. *RR* relative risk

The term “benign breast disease” encompasses a heterogeneous group of non-malignant lesions (Fig. 1) [1–5]. With the introduction of population-based mammographic screening programs, there has been an increased detection of these putative precursor lesions. While the detection of invasive ductal carcinoma (IDC) by mammographic screening programs has increased 1.6-fold, the detection of benign lesions has increased two- to four-fold [6], indicating that not all precursor lesions will ever progress to malignancy. Indeed, a recent study with a median of 12 years follow-up showed that only a minority of women (143 among 698; 20%) with atypical hyperplasia (AH; both atypical ductal hyperplasia (ADH) and atypical lobular hyperplasia (ALH)) eventually progressed to malignancy even without any preventative strategies [7]. Based on their study and other available data the authors concluded that atypical hyperplasia confers an absolute risk of subsequent breast cancer of 30% at 25 years of follow-up [7]. However, the studies shown in Fig. 1 [1–5] show some heterogeneity in the rate of a subsequent breast carcinoma event after a benign biopsy. The variability of associated risk with each type of lesion could be due to multiple factors, including sample size (range 24–336 AH diagnoses), length of follow-up (range 8–17 years), inclusion criteria (e.g., the Nashville study [1] excluded patients who had breast cancer within 6 months of the first biopsy), and in particular the criteria used for atypical hyperplasia diagnosis, which is known to vary as discussed below. Given that each lesion will have variable potential for carcinoma development [5] (Fig. 1), it is important to know which ones are true precursors of breast cancer to facilitate appropriate management. Although both forms of AH, ductal and lobular, carry a similar long-term risk of developing metachronous carcinoma [8, 9], in this review we will particularly focus on ADH, which is histopathologically distinct from ALH [3], presents a more common clinical issue, and has a higher upgrade rate [7].

ADH is not only a risk factor for IDC, it is also considered to be a direct but non-obligate precursor to carcinoma [1]. Diagnosis of ADH carries a four- to fivefold increased risk of developing breast cancer within 5 years that is not limited to the ipsilateral breast [1, 10]. However, Hartmann et al. [7] pointed out that risk estimation has not been calculated as cumulative incidence by the current breast cancer risk prediction tools (such as Gail/Breast Cancer Risk Assessment Tool, International Breast Cancer Intervention Study (IBIS)), and that the lifetime incidence is 25–30% according to multiple large retrospective studies, each with more than 300 AH (both ductal and lobular) diagnoses [7–9, 11].

Strikingly, the risk associated with ADH is doubled with family history, suggesting inherited factors are associated with ADH development. Hoogerbrugge et al. [12] showed that high risk histopathologic lesions, including ADH (39%), were detected in almost 50% of the women younger than age 40 years with a hereditary predisposition for breast cancer who underwent prophylactic mastectomy, not limited to only *BRCA* mutation carriers.

The occurrence of ADH in the general population varies widely from 3% of benign biopsies [13] (based on 30,953 cases), to 8–10% [14, 15] (*n* = 3532), to 23% [16] (*n* = 2833).These differences could come from the total number of biopsies analyzed and/or when these biopsies have been performed (pre/post-widespread mammographic screening). In the pre-mammographic era, biopsies would likely have been performed only for palpable lesions with concomitant low frequency of ADH (2.1% [17], *n* = 10,542). In the current era of mammographic screening biopsies are additionally performed based on micro-calcifications, for example; therefore, a higher frequency of ADH could be observed [18]. These differences may also reflect geographical or temporal differences in incidence, as ADH is associated with hormone replacement therapy (HRT), use of which varies widely over time [13]. Moreover, the variations in ADH frequency may suggest under- or over-diagnosis of ADH at different centers due to differing definitions or variation arising as a consequence of the number of slides sectioned per specimen. For example, Page et al*.* reported only 2.1% ADH in their cohort with, on average, one to five slides per specimen (total original biopsies *n* = 10,542) [17] as opposed to 26 slides by de Mascarel et al*.* (23% detection rate) (*n* = 2833) [16]. In autopsy studies in the general population, ADH was observed in the breast of 3–13% of women [19], which could well be an underestimate, given limited sampling techniques.

In this review, we focus on the definition, diagnosis, and current management of ADH as well as its molecular alterations. We also mention the strengths and limitations of some previous studies and propose ideas for studies that need to be undertaken in order to better understand breast cancer development associated with ADH.

### Definition of ADH

**Fig. 2:**
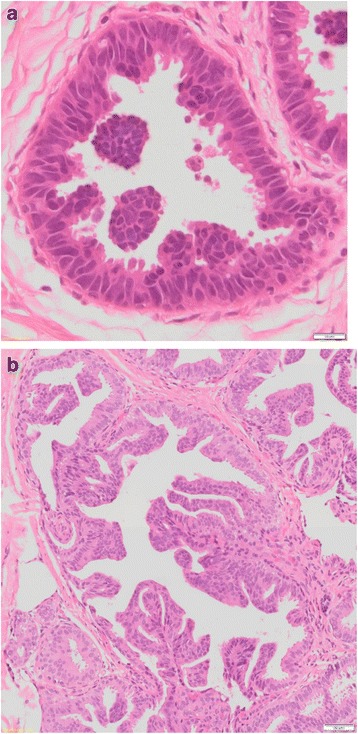
Histological appearance of atypical ductal hyperplasia (40×) (**a**) and low-grade ductal carcinoma in situ (40×) (**b**)

ADH resembles low nuclear grade ductal carcinoma in situ (DCIS) with cytonuclear and architectural atypia but with either partial involvement of the ducts and/or small size for a diagnosis of DCIS. In ADH there are ducts partially filled with abnormally uniform evenly spaced cells with polarization [20] (Fig. 2). ADH and low nuclear grade DCIS (LG DCIS) show not only morphological similarities, including cytological and architectural features, but also immuno-phenotypical overlap (both are estrogen receptor (ER)- and progesterone receptor (PR)-positive and HER2-negative) and especially genomic alterations [21]. ADH has been defined as having “some but not all the requisite features of DCIS” with an involvement of ducts by an architecturally complex proliferation of monotonous cells forming cribriform-like and/or micro-papillary formation with a maximum of two separate spaces [20, 22]. The cells might grow in arcades, rigid bridges, or bars of uniform thickness, micro-papillae, solid or cribriform patterns. The involved spaces might also contain a population of cells with similar characteristics of usual ductal hyperplasia (UDH) or residual normal epithelium [22]. Due to their similar morphological features, ADH and LG DCIS are difficult to reproducibly distinguish; therefore, one other feature was added to differentiate between them—ADH is arbitrarily defined as having a size of ≤ 2 mm [10, 20–22]. Unlike intermediate and high grade DCIS, ADH typically lacks central necrosis and significant nuclear atypia [1].

One of the major impediments to proper management of ADH is the conflicting definitions of ADH and intra-observer variability which make a definitive diagnosis difficult [10, 23]. Ghofrani et al. [23] described instances of the inconsistencies and disagreements of ADH diagnosis among 230 pathologists. For example, one of the scenarios showed that although ADH is defined by having a diameter ≤ 2 mm or only partially involved ducts, when five ducts, each ≤ 2 mm, were involved, 37% of pathologists diagnosed it as DCIS and when > 20 partially involved ducts were involved more than 60% of pathologists diagnosed it as DCIS and recommended excision [23]. Multiple other studies showed a similar lack of concordance in differentiating ADH from LG DCIS among pathologists, ranging from 58 to 92% (reviewed by Walia et al. [10]). However, when standardized published criteria were followed, particularly with a set of provided teaching slides, the concordance rate was satisfactory (71–92%, six participants) [24]. Moreover, one study with nine participants showed not only lack of concordance between ADH and LG DCIS but also between ADH and UDH; however, this poor concordance can be improved by including data from immuno-histochemical staining with multiple cytokeratins (CK) [25]. Particularly, basal type cytokeratins (CK5/6) were found to be very effective for such differential diagnosis [22, 26]. Low nuclear grade neoplasia in the breast, including ADH, typically shows diffuse strong nuclear ER positivity and lacks high molecular weight cytokeratins, such as CK5/6 expression, in keeping with proliferation of end-differentiated luminal cells, unlike UDH (heterogeneous mosaic pattern). Myoepithelial-specific markers such as p63 and myoid markers such as SMA are helpful to demonstrate preservation of myoepithelial cells at the epithelialstroma interface. Notably, while myoepithelial marker positivity in the proliferative pools is useful in the diagnosis of adenomyoepithelioma and other myoepithelial lesions, this does not aid in the differential diagnosis of epithelial hyperplasia versus epithelial neoplasia/atypia [27]. It is important to note that CK5/6 cannot distinguish ADH from LG DCIS as both show uniformly negative staining for luminal cells [25]. Collectively, these studies suggest that a more objective biomarker for the differential diagnosis of ADH and LG DCIS would be highly desirable since extent alone cannot differentiate an early neoplastic lesion that may not progress to malignancy from those that represent part of a DCIS process that is underrepresented in the examined specimen and are more likely to progress. Molecular studies could play an important role to identify such biomarkers; however, given the above described lack of concordance at assessing ADH [28], any studies related to ADH need to include a strict review procedure by one or more breast pathologists with sufficient experience before inclusion into the study.

### ADH diagnosis and management

ADH is usually first identified in a core needle biopsy (CNB), and first designated as a “B3” lesion: i.e., of uncertain malignant potential of the breast. The management of patients diagnosed with ADH on CNB varies not only because of the initial biopsy type/size but also because of the variable reported “upgrade rate”. Upgrade of ADH refers to the finding of cancer (DCIS/IDC) in the surgical excision biopsy that was not present in the CNB. One very recent review stated that 22–65% of ADH diagnosed by CNB were upgraded to carcinoma [29]. The surgical excision rate is lower (60%) if the ADH was diagnosed by vacuum assisted biopsy (VAB), since this technique is thought to be more efficient at removing lesion areas than CNB [29]. While most upgrades of ADH are DCIS, upgrades to IDC do happen [30]. In terms of the management of the patients, some clinicians prefer to observe patients diagnosed with ADH. However, this option may put those patients at risk of undertreatment. Given the high upgrade rate, it is not surprising that the majority of clinicians suggest a surgical excision after ADH diagnosis on CNB to rule out concomitant malignancy. For example, if a patient had an area of radiographic abnormality and her CNB showed a focus of atypical cells in keeping with ADH and not sufficient for the diagnosis of LG DCIS, it would be preferable to perform an excision to examine the whole area of abnormality. Because the abnormal radiological mass might be due to a well-developed DCIS, surgical excision could avoid missing a higher risk lesion requiring more intensive treatment [31]. In this type of upgrade scenario, presumably the most likely explanation for missing a higher risk lesion from a CNB would be sampling limitations. This highlights the important clinical utility of identifying robust biomarkers that can distinguish between pure ADH and ADH that is likely to be associated with a synchronous LG DCIS and thereby avoiding the need for recommending surgical excision for all. Alternatives to surgical excision include treatment with tamoxifen as this has been reported to reduce the risk of developing breast cancer from 21 to 7.5% in 10 years (from 2459 ADH diagnoses) [32]. However, recent studies on breast cancer prevention with endocrine therapy show a very low rate of uptake and even lower rate of persistence due to the side effects, even in women at very high genetic risk [33].

Some parameters may be useful to take into consideration before excision, such as the number of cores, type of needle used, type of lesion, and lesion diameter [7, 10]. It has also been suggested to take into account the number of the foci in the core and how much of the radiographic lesion was removed, with multiple foci requiring a greater area to be removed [34]. Similarly, another recent paper [35] suggested that the radiographic presence of residual lesions after CNB and the maximum lesion size could be predictive of upgrades with 78% sensitivity and 80% specificity. However, this study had only a small and retrospective sample cohort (*n* = 151) and included only those patients already recommended for surgical excision. None of these parameters or suggestions is clinically proven and prospective validation is required to evaluate such prediction tools. Thus, the recommendation and current clinical practice is to perform an open surgical excision on all ADH diagnosed on CNB or VAB unless ADH is a single focus [29, 36]. This practice would certainly overtreat the majority of women diagnosed with ADH and clearly demonstrates the need to identify a robust biomarker to avoid unnecessary surgery and optimal management.

### Risk prediction following ADH diagnosis

Unfortunately, risk prediction following ADH diagnosis is controversial [7], and counseling and further screening for these women diagnosed with ADH are therefore probably not adequate. Indeed, Degnim et al. [8, 11] showed that the 25-year risk of developing cancer associated with ADH is at least 25%, and it could be as high as 50–60% if the ADH is both multifocal and calcified (*n* = 331). One possibility is that this combination indicates a lesion already DCIS at a cellular biology level but lacking the extent to be diagnosed as such. Calcification in their study was present in 70% of the ADH cases, and calcification alone didn’t have a significant independent association with higher risk [8, 11]. An elevated risk of breast cancer associated with calcification of atypical hyperplasia was, however, reported by Hutchinson et al*.* (*n* = 210) [37]. In terms of multifocality, a very recent case-control study from Nurses’ Health Studies found that multifocality was only a significant risk factor for ALH (*n* = 110) rather than ADH (*n* = 173) [3]; differences in sampling and the level of centralized pathology review between the two studies are potential confounders for these observations. Although the multiple foci of ADH were well defined by the pathologists to distinguish between ADH and LG DCIS in Degnim et al*.* [8, 11] and the Nurses’ Health Studies [3], the location of calcification was not specified (intra-ductal versus stromal) [8, 11] or was unknown [3]. As calcification is one of the common features of ADH [16, 22], it would have been really interesting to see if intra-ductal calcification and/or stromal calcification correlate with different risk. The degree of atypia can also vary in ADH, but this is not a feature that has been evaluated in terms of subsequent risk, perhaps because of the difficulty in standardizing such a measure across different pathologists.

Apart from the histologic variables, younger/pre-menopausal women (< 45 years) are at higher risk of developing metachronous carcinoma as found by several studies [8, 38, 39], particularly higher grade cancer [40]. The complexities of analyzing focality, calcification, and atypia in terms of how these are measured emphasizes the importance of understanding the breast biology, as well as the precursor versus risk indicator status of ADH. Given the fact that only a fraction of ADH (9.8–30%) [7, 41] develop metachronous carcinoma, a molecular marker of risk may have the potential to be more objective than clinical symptoms or histopathological features alone for the management of patients.

Thus far, despite the high risk of developing cancer associated with ADH, attempts to identify clinicopathological or molecular biomarkers to predict individual risk have been unsuccessful. Risk reduction strategies remain varied, from active surveillance at one end of the spectrum to prophylactic mastectomy at the other.

### Molecular features of ADH

Breast cancer is well known to be a genetic disease, with very frequent somatic copy number changes, a number of driver mutations such as in *PIK3CA* and *TP53*, and widespread transcriptional deregulation [42]. While molecular studies of benign breast disease are fewer, they suggest both similarities to and differences from breast carcinoma.

### Genetic events in pure ADH

Very few studies have described the molecular genetic features of ADH (Table 1) and these are further limited because most were carried out on small numbers of samples using low resolution methodology, such as microsatellite marker-based loss of heterozygosity (LOH) or allelic imbalance (AI) analysis and cytogenetic comparative genomic hybridization (CGH). The latter has a genomic resolution of 5–10 Mb, whereas most LOH studies were carried out using only a few markers, which were chosen based on the location of common regions of AI in IDC. One of the major barriers to studying ADH is the limited amount of DNA available, a problem reported by multiple studies [43, 44]. This limitation can be overcome by using in situ assays, such as fluorescence in situ hybridization (FISH), but at the cost of being highly locus-specific.

**Table 1:** Major genetic features of atypical ductal hyperplasia

Method^a^	Number of samples	Number of loci or genomic resolution	Cases with aberration	Average cases altered per locus	Location of copy number gain^a^	Location of copy number loss/LOH/AI^a^	SNV^a^	Reference
LOH	10^P^	2	50%	38.9%	NA	16q, 17p	NA	[43]
LOH	26^P^ 25^S^	15	42%^P^ 44%^S^	6.2% 9.5%	NA	11p, 13q, 16q, 17p, 17q	NA	[45]
LOH	23^S^	14	NA	15%	NA	8p,16q,17q	NA	[79]
LOH	16^S^	22	75%	13%	NA	1q, 3p, 11p, 11q, 16q, 17p	NA	[44]
LOH	31^P^	26	65%	6.1%	NA	8q	NA	[67]
LOH total	131 (67^P^, 64^S^)	2–26	53%^P^, 70%^S^	15%	NA	16q (24%), 13q (15%), 17q (12%), 11p (12%), 17p (10%)	NA	
CGH	9^P^	5–10 Mb	55%	NA	1q, 16p, 11q	16q, 17p, 20p	NA	[80]
CGH	2^P^	5–10 Mb	100%	NA	1q, 3p, 6p, 10p, 11q, 12q, 13q, 16p, 17q, 20q, 8q, 14q, 15q	4q, 5q, 1p, 13q, 16q, 17p	NA	[55]
CGH	3^S^	5–10 Mb	100%	NA	3p, 8q, 15q, 16p, 20q, 22q	13q, 16q	NA	[56]
CGH	15^P^	5–10 Mb	93%	NA	1p, 1q, 2q, 8q, 10p, 17q, 20q, 20p, 2q, Xp	8p, 9p, 11q, 13q, 14q, 16q, 21q, Xp	NA	[54]
CGH total	29		80%^P^, 100%^S^	NA	8q, 20q, 16p, 17q, 1q	16q, 13q, 17p, 8p	NA	
Targeted sequencing	4	130–296 SNV/case	100%	NA	NA	NA	Lineage heterogeneity	[58]
WGS	2	1 base pair	100%	NA	1q gain early neoplastic event	–	ADH and carcinoma shared SNVs	[57]
FISH^P^	9	8	100%	45.6%	7, 8, 18	–	–	[51]
FISH^S^	13	1	54%	NA	Higher *ERBB2* amplification from ADH to DCIS to IDC	NA	NA	[65]

^a^*LOH* loss of heterozygosity, *AI* allelic imbalance, *CGH* comparative genomic hybridization, *SNV* single nucleotide variant, *WGS* whole-genome sequencing, *FISH* fluorescence in situ hybridization, *NA* not available or not applicable. Gains are considered when chromosomal imbalance is > 1.25 and losses are considered when it is < 0.8 of the normal allelic ratio. AI is considered when the imbalance is > 1.33 or < 0.75 of the normal allelic ratio. Any gains or losses are reported when changes occurred in at least one sample of the cohort. *P* = pure ADH (no synchronous carcinoma), *S* = ADH with synchronous carcinoma

An LOH study of 41 ADH samples at 15 genetic loci selected based on the locations of frequently inactivated tumor suppressor genes in IDC, such as *TP53*, *RB1*, and *BRCA1*, reported that 42% of “pure” ADH (without synchronous DCIS/IDC) showed LOH in at least one locus, suggesting that inactivation of these tumor suppressor genes might be an early neoplastic event and related to the subsequent development of IDC [45]. This study identified loss of 16q as an LOH “hot spot” in ADH and also in low-grade DCIS and IDC. Loss of 16q was also confirmed as a common event in breast cancer by other studies (Table 1), signifying that this event might be particularly important in the early development of breast cancer. Indeed, flow cytometry studies have suggested that 15–44% of ADH are aneuploid, indicating that copy number alterations are often present at this pre-malignant stage [46–50]. A more sensitive FISH study using nine chromosomal probes supported these observations, finding that all ADH had chromosomal aneuploidy, and the number of cells with aneuploidy was higher than in lesions without atypia, although less frequent than in carcinomas [51]. In contrast, two other FISH analyses observed aneuploidy in 1/5 and 0/2 ADH using two and six probes, respectively, whereas carcinomas all carried aneuploidies [52, 53].

Using alternative methods, Gao et al. [54] detected copy number aberrations in 15 pure ADH samples by array CGH and found that although there were similar genetic alterations among ADH, DCIS, and IDC, there were also alterations unique to each lesion. Firstly, they found that gain at 19p and losses at 2p, 6q, 11p, 12q, 22q, and Xq were only present in DCIS and IDC, suggesting that these changes might be a later event in breast cancer progression. Secondly, ADH had a high prevalence of 17q gain [54], although the number of cases studied was small. In addition, this study is the only one in the literature in which ADH showed more copy number changes than DCIS. This unusual feature, along with the pattern of CGH alterations with a high proportion of changes at telomeres, may suggest an imperfect assay, particularly for ADH samples where material would have been limited. Two other CGH studies each showed several copy number gains (Table 1); however, the sample sizes were just two (pure ADH) [55] and three (synchronous) [56].

These genetic studies emphasize the difficulty in analyzing small numbers of cases with varying definitions of ADH, often leading to different conclusions. Nonetheless, despite individual small sample sizes, across all LOH and CGH studies, 16q loss remained the most common cytogenetic event in pure ADH, followed by 1q gain, with other loci being gained and lost at relatively lower and varying frequencies. In addition, collectively these studies show that most pure ADH carry one or more large-scale cytogenetic abnormalities.

### ADH as a precursor lesion

As well as evaluating pure ADH, a common strategy has been to study “synchronous” ADH found in the same breast as carcinoma (DCIS or IDC). The goal of these studies has been to establish whether ADH could be a genetic precursor to carcinoma (and determine which type of carcinoma), and to evaluate whether genetic events are required for progression.

Early studies used LOH to investigate the clonal relationship between ADH and associated cancers; for example, Larson et al. [44] performed a microsatellite analysis of 45 ADH samples with co-existing DCIS or IDC from 16 patients. These studies found that ~ 60% of the cancer cases had a co-existing clonal ADH lesion. More recently, next-generation sequencing approaches have been applied, although only in small numbers of cases so far. For example, Newburger et al. [57] showed that atypical hyperplasia shared a common ancestor with the carcinoma based on somatic mutations, although this was based on only two patient samples [57]. Similarly, Weng et al. [58] assessed the phylogenetic relationship between early neoplasias, including ADH, and DCIS and IDC by studying six breast cancer patients with concurrent neoplasias (four with atypia). This analysis revealed considerable lineage heterogeneity and the authors suggested that the early neoplastic lesions and DCIS were not direct precursors of the co-existing IDC, but rather independent clonal proliferations of cells with a common ancestor. Interestingly, they also found that some neoplasias showed a mixed-lineage origin, referring to the samples whose cells are geographically co-located but have originated from at least two genetically diverse lineages (although often still sharing an ancient common ancestor). The accumulated somatic mutations of those samples could not be explained by a single lineage tree, suggesting existing high intra-individual genetic heterogeneity, which was also observed by Larson et al. [44]. Given that genetic heterogeneity may be a bad prognostic feature in several tumor types [59], its detection in benign lesions could be relevant for patient management. If multiple neoplastic or atypical lesions were detected by high resolution imaging, phylogenetic analysis of carcinoma and concurrent neoplasia/ADH might change the direction of the patients’ management by treating or removing not only cancerous lesions, but also the reservoir of genetically diverse neoplasias to prevent recurrence.

These genetic studies to date support a role for ADH as a precursor of carcinomas identified in the same breast, but they do not explain the risk associated with ADH for the contralateral breast. It has been noted in multiple studies that ipsilateral recurrence is most common (almost twice that of contralateral) in the first 5 years after ADH diagnosis; however, the long-term risk remains high for both breasts [7, 9]. It is important to note that ipsilateral recurrence is not limited to the initial site of diagnosis of ADH and in cases where the carcinoma recurs at a different quadrant, it could be speculated that this would be non-clonal. Indeed, this possibility is supported by Larson et al., who noted that ADH in different tissue blocks to the co-existing carcinoma were less likely to be clonal (75% clonal when in the same block vs 27% when in different blocks) [44]. Interestingly, they also observed the presence of ADH heterogeneity in 46% of cases when there were multiple ADH foci in the same cancer-containing breast, indicating independent origins of the lesions. ADH may therefore also be a marker of elevated risk not associated with clonal recurrence. As suggested by a study of field cancerization effect in epidermoid carcinoma [60], ADH and other benign lesions could be the result of a “field effect” where non-related tumors are co-located within a cancer-prone tissue. Apart from germline predisposition, the cause of such a field effect in the breast is not yet known; however, at least for ER+ tumors, factors suggested by epidemiological studies could play a role—for example, parity, breast feeding, and mammographic breast density [3]. Other environmental risk factors, such as alcohol consumption, smoking, or obesity, could also contribute to such a “field”. These micro-environmental influencers could provide a possible explanation for the initiation of multiple breast lesions over long periods of time, their persistence, and their progression to carcinoma. Particularly, ADH in younger women could be the result of an oncogenic insult and/or extreme susceptibility for the proposed oncogenic estrogen metabolites associated with the premenopausal hormonal environment [8]. Studying the association of atypia with these characteristics could give an insight into identifying patients with a higher risk of recurrence. Interestingly, a very recent study showed no association between mammographic breast density and risk of recurrence in patients diagnosed with atypical hyperplasia [14]. Breast density, therefore, despite being a major indicator of an altered breast microenvironment, appears not to influence subsequent progression to carcinoma after ADH. Similarly, higher BMI, early menarche, and smoking are not associated with a higher risk of developing invasive cancer after a previous breast benign biopsy [61]. Further study is needed to evaluate the different contributions of these factors for disease initiation as distinct from disease progression. The role of the immune system has barely begun to be investigated as a factor controlling disease progression, but could well be crucial.

Early models of breast cancer development, which proposed a direct linear progression from normal epithelium to ductal hyperplasia to ADH to low-grade DCIS and then to low- or high-grade IDC, are now considered to be oversimplified [21]. Instead, distinct low- and high-grade multistep models of breast cancer progression have been hypothesized [21]. The “low-grade like” progression pathway is characterized by recurrent loss of 16q (>  75%), gains of 1q; expression of hormone receptors (ER+, PR+), lack of HER2 overexpression and a low-grade-like gene expression signature [21, 62, 63]. The “high-grade-like” progression pathway is characterized mainly by gains of 8q (75%) and 1q (60%), losses of 1p (60%), 8p (60%), and 17p (60%), and a luminal B, HER2, or basal-like mRNA expression profile [21, 63]. Studies of breast cancer stem cells also suggest that, apart from the claudin-low subtype, the cell of origin for the other intrinsic breast cancer subtypes may originate at different points along the luminal progenitor lineage [64]. It remains unknown if distinct precursors arise from these progenitors since many of the molecular alterations are not necessarily exclusive to each pathway. Where does ADH fit into this new paradigm?

Regarding ADH progression, a prevailing view is that ADH is only a direct precursor of LG and ER-positive carcinoma [21]; however, this is not supported entirely by the literature. On one side, Larson et al. demonstrated that clonality between ADH and synchronous carcinoma was more likely when the carcinoma was low grade and that ADH lacking any AI was most commonly associated with high grade cancer, although these trends were non-significant [44]. However, at least two of the clonal cases studied by Larson et al. must have been of high grade, although this was not explicitly stated. Indeed, few genetic studies have stated the grade of cancer synchronous to ADH. In addition, the later development of breast carcinoma associated with ADH is not limited to LG cancer. Two recent studies [9, 40] showed that about two-thirds of the recurrent breast carcinomas were ER+ intermediate/high grade. It is noteworthy that small subsets of patients diagnosed with ADH developed ER− (9%) and/or HER2+ ductal carcinoma (7%) [40], including ipsilateral recurrences. As the precursor of HG DCIS and/or HG IDC is still unknown, a synchronous ADH genomics study with LG and HG carcinoma and including all intrinsic subtypes of carcinoma, along with detailed histopathological features of the ADH, would be highly desirable to determine the precursor relationship. It would certainly aid in patient management if we could identify the subsets of patients diagnosed with ADH that might develop HG cancer and treat them accordingly. In fact, after the accurate diagnosis of ADH, this question is one of the most challenging unaddressed clinical questions regarding ADH. Degnim et al. [8] showed the importance of both the number of foci of ADH and calcification as features associated with a higher risk of recurrence; however, they did not mention whether any of these features also significantly correlated with grade or ER status of the subsequent breast carcinoma. Correlative studies of tumor type after ADH also do not address the genetic relationship of recurrences to previous ADH: at present, this is entirely unknown, but critical in order to understand the natural history of ADH and to guide therapy choices.

While genetic analysis of ADH with high-grade and/or ER− carcinomas is underrepresented in the literature, HER2 cases have been addressed through the use of FISH. Interestingly, a FISH study of synchronous cases reported that the amplitude of the amplification of *ERBB2* increased from ADH to DCIS to IDC [65]. Fifty-four percent of ADH synchronous with HER2+ IDC showed low or moderate *ERBB2* amplification, suggesting *ERBB2* amplification can be involved early in breast oncogenesis but higher amplification may be required for progression [65]. This result supports ADH as a precursor lesion for HER2+ cancer, consistent with the observation of HER2+ breast cancer arising after an ADH diagnosis [9, 40].

Other studies of ADH synchronous to carcinoma have also attempted to identify genetic events associated with progression, similar to studies comparing pure DCIS to DCIS synchronous with IDC, in which it has been observed that pure DCIS have different molecular profiles to synchronous DCIS, with the latter carrying more copy number changes overall [66]. Similarly, when pure ADH was compared to synchronous ADH, pure ADH showed less AI compared to cases synchronous with DCIS or IDC, although the power of the studies was limited [45, 67]. However, while DCIS was genetically very similar to synchronous IDC [68, 69], the overlap between synchronous ADH and carcinomas has shown that even when clonally related (~ 60% of the time) co-existing carcinomas often have additional genetic events [44]. In addition, a sequencing study found that while few driver point mutations were found, patients with atypical hyperplasia shared aneuploidy events with the carcinomas, suggesting that copy number change, particularly the 1q gain commonly observed in IDC [70], might be an early driver of the neoplastic phenotype [57]. Their findings also suggested that early neoplasias can harbor sufficient driver aneuploidy events to progress into carcinoma, possibly with a combination of mutational load and accumulated aneuploidy, as well as epigenetic and stromal changes over time. A similar aneuploidy hypothesis was proposed by Forsberg et al. [71], who observed copy number changes in histologically normal epithelial cells at uninvolved margins of IDC. However, these studies only included cases with synchronous carcinoma, which may not be representative of atypical hyperplasia without co-existing carcinoma. Overall no consistency in specific genetic events can be attributed to progression. This may reflect inter-tumoral genetic heterogeneity and/or that the number and combination of drivers are more important than the order of genetic events. It may not be possible, therefore, to map specific genetic events to the cancer phenotype, although as noted already, the number of cases in the current literature is inadequate to say whether this is indeed the case.

### Transcriptional changes in ADH

As well as the genetic events described above, progression to IBC from ADH may be evaluated by gene expression differences, which can also reflect the influence of the local environment. In order to understand the key driver events in breast cancer progression, Brunner et al. [72] carried out expression analysis of matched normal, ADH, and cancer tissue from 16 patients to characterize transcriptional differences. Interestingly, they found a pro-oncogenic gene expression signature in early neoplasia which was distinct from normal tissues and carcinoma (DCIS/IDC) including up-regulation of *ERBB2*, *FOXA1*, and *GATA3* [72]. The *ERBB2* mRNA overexpression was not thought to be due to genomic amplification of the *ERBB2* locus since only three cases tested clinically positive for HER2 amplification in the IDC. They suggested that *ERBB2* has a role in early stages of breast cancer development independent of gene amplification [72]. However, this conclusion is not supported by two other immunohistochemistry-based studies, which did not identify any overexpression of *ERBB2* among 44 and 19 atypical hyperplasias, respectively [73, 74]. The prognostic and predictive factors of *ERBB2* amplification and/or overexpression should be studied extensively in a larger cohort. *GATA3* up-regulation was highly correlated with ER positivity and *FOXA1* expression in this study [72]. As *FOXA1* is one of the early events in the ER pathway activation cascade, it might be possible that the oncogenic nature of ER pathway activation is already established in early neoplasia and continues to IDC as *FOXA1* and *GATA3* are frequently mutated in ER+/luminal breast tumors [42]. Additionally, Brunner et al. [72] reported that several pathways influencing membrane transport, including endocytosis by ABC transporters, fatty acid metabolism, and phenylalanine metabolism, are highly enriched already in early neoplasia compared to normal tissues. Notably, these pathways do not encompass any well-known oncogenes; thus, they should be explored further to elucidate the mechanisms involved.

A second gene expression profiling study of ADH synchronous with cancer (DCIS/IDC) (*n* = 31, eight with ADH) showed that significant alterations are already present in ADH and maintained in DCIS and IDC [75]. All ADH showed a grade 1 gene expression pattern and clustered with low grade DCIS and IDC, confirming the close relationship between ADH and low-grade carcinoma (DCIS/IDC) and that ADH have potential to progress into carcinoma [75]. Interestingly, *GATA3* was differentially expressed in this study, which supports the finding of Brunner et al. [72]; however, *FOXA1* and *ERBB2* were not reported. In addition, other key differentially expressed genes and pathways found in Brunner et al., such as the ABC transporters, were not found in Ma et al. (except *ABCA8* but with a low enrichment) [75]. Only around 60 genes overlapped between these two studies; however, there is a very poor correlation of gene expression profiling reported previously between microarrays and RNA sequencing with formalin-fixed paraffin embedded tissues [76]. In general, however, both studies showed that ADH was clearly different from normal breast epithelium, and additional differences were noted on progression to carcinoma.

While these studies are informative for ADH present in synchrony with carcinoma, the expression profiles of pure ADH have not been adequately assessed in a sufficiently powered study. One study did attempt to profile pure ADH in comparison to ADH associated with carcinoma; however, the tissue used was taken adjacent to the ADH lesion observed histologically, with no certainty that the ADH lesion was in fact present [77]. Nonetheless, some overlap was observed with genes differentially expressed in Ma et al. [75] and the authors proposed MMP-1 as a biomarker for progression to carcinoma. A detailed transcriptional study with a larger cohort consisting of pure ADH with extensive patient outcome data would be very powerful in order to identify new pathways for breast cancer prevention associated with ADH. Such studies are increasingly becoming feasible, as the technology for transcriptional studies from formalin-fixed, paraffin-embedded tissue becomes more robust.

## Conclusions

The increasing diagnoses of ADH as a consequence of population-based mammographic screening have created clinical dilemmas for treating physicians. Should ADH always be excised or are other options viable? Understanding the genetics of ADH might lead to effective strategies to prevent development and progression of breast cancer associated with ADH and shed light on the breast cancer progression model, in particular the relationship of ADH with non-low grade as well as ER− carcinoma. In addition to synchronous cases, cases with neoplasia not associated with cancer should be assessed in depth as these could be informative for early diagnosis and preventative therapeutic strategies. The comparison of pure ADH with synchronous ADH and cases where ADH was upgraded to carcinoma on excision may be informative for development of biomarkers to help aid in clinical treatment decisions. Overall, the various limitations of all the previous studies discussed in this review (small sample size, lacking careful selection of ADH with and without carcinoma, low resolution methodology, etc.) need to be overcome in any future study of ADH. With the improvement of next-generation sequencing technologies, a careful selection of a larger cohort of ADH than studied to date (with and without carcinoma of different grades), reviewed by an experienced pathologist, would give an insight into early breast cancer progression. Cases of ADH with at least 25 years follow-up should also be included to differentiate between the cancerized and non-cancerized lineages [78], whereby the former is the subset of ADH that could progress to carcinoma while the latter subset would lack progression capability even when harboring clonal genetic events. The outcome of such a study could reduce the burden of overtreatment associated with ADH.

## Abbreviations

ADH: Atypical ductal hyperplasia
AI: Allelic imbalance
CGH: Comparative genomic hybridization
CNB: Core needle biopsy
DCIS: Ductal carcinoma in situ
ER: Estrogen receptor
FISH: Fluorescence in situ hybridisation
IDC: Invasive ductal carcinoma
LG DCIS: Low-grade DCIS
LOH: Loss of heterozygosity
PR: Progesterone receptor
VAB: Vacuum assisted biopsy
WGS: Whole genome sequencing
